# Mitochondrial Transfer Regulates Cell Fate Through Metabolic Remodeling in Osteoporosis

**DOI:** 10.1002/advs.202204871

**Published:** 2022-12-11

**Authors:** Wenjin Cai, Jinglun Zhang, Yiqian Yu, Yueqi Ni, Yan Wei, Yihong Cheng, Litian Han, Leyi Xiao, Xiaoxin Ma, Hongjiang Wei, Yaoting Ji, Yufeng Zhang

**Affiliations:** ^1^ State Key Laboratory Breeding Base of Basic Science of Stomatology (Hubei‐MOST) and Key Laboratory of Oral Biomedicine Ministry of Education School and Hospital of Stomatology Wuhan University Wuhan 430079 China

**Keywords:** intercellular mitochondria transfer, macrophages, mesenchymal stem cells, metabolic bone diseases, mitochondria

## Abstract

Mitochondria are the powerhouse of eukaryotic cells, which regulate cell metabolism and differentiation. Recently, mitochondrial transfer between cells has been shown to direct recipient cell fate. However, it is unclear whether mitochondria can translocate to stem cells and whether this transfer alters stem cell fate. Here, mesenchymal stem cell (MSC) regulation is examined by macrophages in the bone marrow environment. It is found that macrophages promote osteogenic differentiation of MSCs by delivering mitochondria to MSCs. However, under osteoporotic conditions, macrophages with altered phenotypes, and metabolic statuses release oxidatively damaged mitochondria. Increased mitochondrial transfer of M1‐like macrophages to MSCs triggers a reactive oxygen species burst, which leads to metabolic remodeling. It is showed that abnormal metabolism in MSCs is caused by the abnormal succinate accumulation, which is a key factor in abnormal osteogenic differentiation. These results reveal that mitochondrial transfer from macrophages to MSCs allows metabolic crosstalk to regulate bone homeostasis. This mechanism identifies a potential target for the treatment of osteoporosis.

## Graphical abstract

The findings reveal a new pattern of cellular crosstalk in the bone marrow environment, with macrophages regulating bone metabolism crosstalk by transferring their mitochondria into mesenchymal stem cells. This study suggests that mitochondrial transfer may be a novel way to regulate homeostasis by enabling immune cells to regulate their local tissue environment.

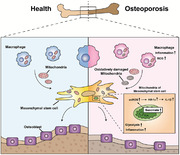

## 1 Introduction

Coordinated activity of various cell types in the bone marrow is essential for maintaining homeostasis.^[^ ^1^ ^]^ Bone homeostasis is mainly determined by the balance between bone resorption by osteoclasts and bone formation by osteoblasts and is closely related to the overall metabolism.^[^ ^2^ ^]^ Compared to healthy people, patients with metabolism‐related diseases caused by factors, such as age and hormones have disrupted bone homeostasis and are at higher risk of osteoporosis and fragility‐related fractures.^[^ ^2^, ^3^ ^]^

Osteoporosis is a systemic metabolic disease characterized by imbalanced bone homeostasis, low bone mass, and microstructural changes. Previous studies showed that immune cells, such as macrophages, are essential for maintaining bone homeostasis and are involved in the development of osteoporosis.^[^ ^4^ ^]^ Macrophages are a heterogeneous population of cells that are converted to a proinflammatory (M1) or anti‐inflammatory (M2) state in response to environmental stimuli. These cells regulate bone homeostasis by secreting related factors.^[^ ^4^, ^5^ ^]^ Macrophages promote osteogenic differentiation of mesenchymal stem cells (MSCs) through osteostatin (OS)‐M and bone morphogenetic protein (BMP)‐2 secretion.^[^ ^6^ ^]^ Interleukin (IL)‐6 and tumor necrosis factor (TNF)‐*α* are secreted by proinflammatory M1‐like macrophages and inhibit osteogenic functions.^[^ ^5^, ^7^ ^]^ Current research on signaling between macrophages and MSCs mainly focuses on how macrophages regulate MSC activity by paracrine signaling. However, in the complex bone marrow environment, whether there are additional direct communication modes needs further study.

Previous studies on intercellular communication focused on electrical coupling, paracrine signaling, and gap junction transmission. Recently, new communication modes that rely on organelle transfer between cells have received considerable attention.^[^ ^8^ ^]^ Mitochondria are the energy source for eukaryotic cells and are crucial for regulating cell metabolism and differentiation. Thus, mitochondrial transfer is currently receiving extensive attention.^[^ ^9^ ^]^ Recipient cells can capture functional mitochondria released by donor cells into the extracellular space.^[^ ^9^, ^10^ ^]^ Tunneling nanotubes (TNTs) and microvesicles (MVs) are the most common mitochondrial transport pathways.^[^ ^11^ ^]^ The form of mitochondrial transfer between cells helps the mitochondria of donor cells integrate into the endogenous mitochondrial network of receptor cells, thus causing changes in the bioenergy status and other functions of receptor cells.^[^ ^12^ ^]^ However, the circumstances under which mitochondrial transfer occurs and the underlying physiological implications require further investigation.

Mitochondrial transfer is implicated in multiple disease processes, including inflammatory pain,^[^ ^13^ ^]^ ischemic stroke,^[^ ^14^ ^]^ hypoxia‐induced pulmonary hypertension,^[^ ^15^ ^]^ acute lung injury repair,^[^ ^10^ ^]^ cancer cell growth,^[^ ^11^, ^16^ ^]^ and allograft rejection.^[^ ^17^ ^]^ Since mitochondrial transfer may be involved in the pathogenesis of some diseases, interventions targeting the transfer process are currently under investigation.^[^ ^18^ ^]^ Spees et al. demonstrated that mitochondria‐depleted cells restore normal cellular viability by capturing healthy mitochondria from donor cells.^[^ ^19^ ^]^ Other studies showed that when cells such as lung epithelium, cardiac muscle, and neurons are damaged, supplementing healthy mitochondria from MSCs can restore mitochondrial homeostasis and energy metabolism of the recipient cells.^[^ ^9^, ^20^ ^]^ However, the metabolic cascade in the target cells that are activated by mitochondrial transfer and the pathological processes intervened by mitochondrial transfer remain unclear. Further, how energy and MSC fate are regulated by other cells during mitochondrial transfer has not been well studied.

The bone marrow in which macrophages and MSCs are present is a freely dispersed environment. Therefore, it is necessary to investigate whether macrophages can transfer mitochondria to MSCs to regulate MSC fate. Here, we aimed to study macrophages that undergo mitochondria‐specific energy stress to release oxidatively damaged mitochondria under osteoporotic conditions and the effect of mitochondria delivery on reactive oxygen species (ROS) production and osteogenic function of MSCs. Furthermore, our results suggest that circulating succinate induces elevated ROS levels and hypoxia inducible factor‐1*α* (Hif‐1*α*) activation to promote increased expression of pro‐inflammatory genes, which in turn affects the osteogenic differentiation of MSCs. Collectively, cell‐to‐cell mitochondrial transfer in the bone marrow environment represents an underappreciated mechanism of cellular communication that has various regulatory effects on bone metabolism and provides new ideas for the prevention and treatment of osteoporosis.

## 2 Results

### 2.1 Macrophages Deliver Mitochondria to MSCs in the Bone Marrow

**Figure 1:**
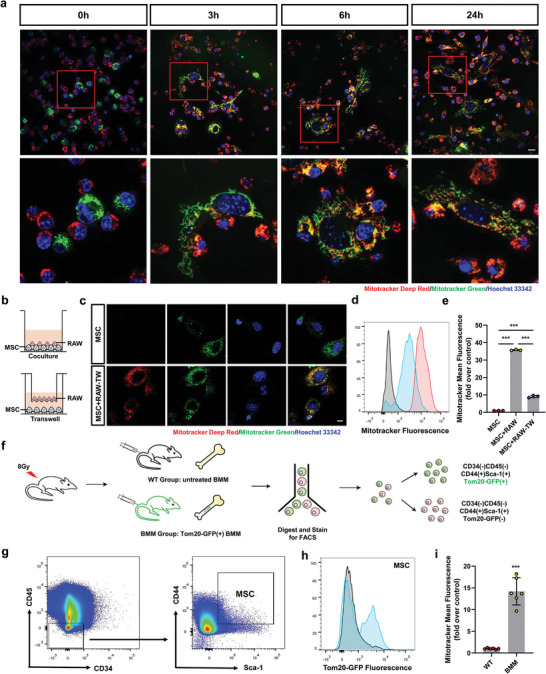
Macrophages deliver mitochondria to MSCs in the bone marrow. a) Representative confocal microscopy pictures of macrophages incubated with MSCs for 0, 3, 6, and 24 h, MSCs were previously labeled with Mitotracker Green (green), and macrophages were labeled with Mitotracker Deep Red (red), Nuclei were labeled with Hoechst 33 342 (blue) (*n* = 3). Scale bar, 10 µm. b) The coculture system and the transwell (TW) culture system determine the mitochondrial transfer form. c) Typical confocal microscope photos of macrophages and MSCs incubated in the TW culture system for 24 h. MSCs were previously labeled with Mitotracker Green (green), and macrophages were labeled with Mitotracker Deep Red (red), Nuclei were labeled with Hoechst 33 342 (blue) (*n* = 3). Scale bar, 5 µm. d,e) Flow cytometric analysis d) and quantification of mean fluorescence intensity e) of macrophages and MSCs incubated in TW culture system for 24 h. Before incubation, macrophages were marked with Mitotracker Deep Red (red). After 24 h of coculture system or the TW culture system, MSCs were collected for flow cytometry to analyze the mean fluorescence intensity of Mitotracker Deep Red (red) in MSCs. Gray line, untreated MSCs. Red line, MSCs exposed to untreated macrophages. Blue line, MSCs exposed to macrophages treated with the TW system (*n* = 3); one‐way ANOVA with Tukey's post‐test. f–i) Schematic representation of mitochondrial transfer assessment in vivo f). Before the tail vein injection of untreated BMM (WT group) or Tom20‐GFP‐labeled BMMs (BMM group), mice received a dose of 8 Gy. Flow cytometry quantified the transfer of mitochondria from Tom20‐GFP‐labeled BMMs to MSCs g) and quantified the mean fluorescence intensity of GFP in MSCs h,i) (*n* = 6). One‐way ANOVA with Tukey's post‐test. Each error bar represents the mean ± SD of three independent experiments. Differences were considered statistically significant at *p* < 0.05. *p < 0.05, **p < 0.01, and ***p < 0.001.

To verify mitochondrial transfer from macrophages to MSCs, we labeled MSCs with MitoTracker Green and cocultured them with macrophages (labeled with MitoTracker Deep Red) for 0, 3, 6, and 24 h. Confocal microscopy and flow cytometry confirmed the presence of macrophage‐derived mitochondria in MSCs (**Figure** **1**a,d,e; and Video S1, Supporting Information). Previous studies found that mitochondrial transfer relies on membrane carriers such as TNTs and MVs in most cases.^[^ ^11^ ^]^ Moreover, free mitochondria or mitochondrial components can also be extruded or internalized into recipient cells. To investigate the mechanism of mitochondrial transfer and acceptance, we analyzed mitochondrial transfer from macrophages (labeled with MitoTracker Deep Red) to MSCs using a transwell (TW) culture system (0.4 µm pore size) to isolate MSCs from macrophages. Mitochondrial transfer from macrophages to MSCs was partially inhibited in TW culture (Figure 1b–e), indicating that free mitochondria or mitochondria‐containing MVs can be internalized by MSCs.

A transmission electron microscopy (TEM) analysis demonstrated that macrophages deliver mitochondria to MSCs in various ways, including free mitochondria, MVs, and TNTs. Further, these structures were internalized by MSCs (Figure S1a,b, Supporting Information). MSC internalization was inhibited by dynasore, an endocytosis inhibitor that acts on dynamin‐dependent clathrinid activity but had no effect on MSC viability (Figure S1c–e, Supporting Information). After preventing the endocytosis of MSCs, the number of mitochondria from macrophages in MSCs decreased significantly (Figure S1c,d, Supporting Information). Finally, we investigated the mitochondrial morphology of MSCs after mitochondrial transfer from macrophages. TEM analysis showed that untreated MSCs had shorter mitochondrial morphology. In contrast, MSCs exposed to macrophages could detect a large number of confluent mitochondria after 24 h of coculture. (Figure S1f, Supporting Information), accompanied by increased expression of Mfn2 in MSCs (Figure S1g,h, Supporting Information).

To test whether intercellular mitochondrial transfer occurs in vivo, we extracted bone marrow‐derived macrophages (BMMs) for cell transplantation experiments. BMMs were labeled with the mitochondrial marker protein Tom20 and used in subsequent experiments after flow cytometry (Figure 1f; and Figure S2a–c, Supporting Information). Confocal microscopy further confirmed the presence of mitochondria of Tom20‐GFP (+) from BMM in MSCs (Figure S2d, Supporting Information). BMMs expressing Tom20‐GFP were re‐injected into irradiated mice via the tail vein (Figure 1f). We analyzed the MSC phenotype using flow cytometry (Figure 1g; and Figure S2e, Supporting Information) and found that green fluorescence appeared in MSCs (Figure 1h,i; and Figure S2f, Supporting Information), indicating that mitochondrial delivery from macrophages to MSCs occurs in vivo.

### 2.2 Mitochondrial Function in Macrophages is Altered in Osteoporosis

**Figure 2:**
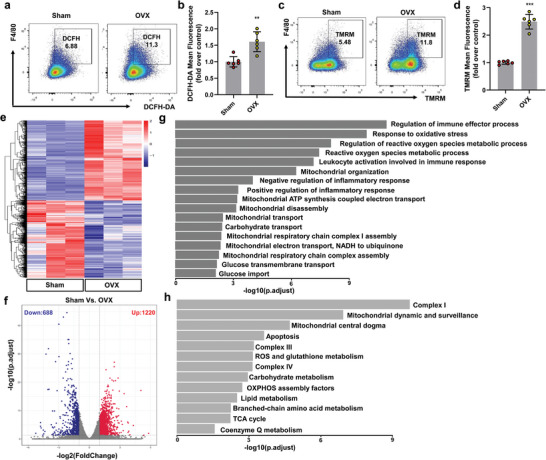
Mitochondrial function of macrophages is altered in osteoporosis. a,b) Macrophages were co‐treated with DCFH‐DA and F4/80 for 30 min, by flow cytometry to quantify ROS and mean fluorescence intensity as a measure of cellular reactive oxygen species production (*n* = 6). Two‐tailed Student's *t*‐test. c,d) Macrophages were cotreated with TMRM (20 nm) and F4/80 for 30 min and then analyzed by flow cytometry to quantify membrane potential (*n* = 6). Two‐tailed Student's *t*‐test. e–h) RNA‐seq analysis in sham or OVX‐treated BMMs (*n* = 3). e) Genes shown by HC. f) Volcano plot of all identified genes. g) GO analysis. h) GO analysis of genes encoding proteins with mitochondrial localization (based on MitoCarta 3.0 dataset) (*n* = 3). Each error bar represents the mean ± SD of three independent experiments. Differences were considered statistically significant at *p* < 0.05. *p < 0.05, **p < 0.01, and ***p < 0.001.

Macrophages are essential for regulating bone homeostasis.^[^ ^4^ ^]^ Moreover, immune cell activation and function are closely related to cellular metabolism.^[^ ^21^ ^]^ To investigate the metabolic profile of macrophages in osteoporosis, we used an ovariectomy (OVX)‐operated mouse osteoporosis model. Using the flow cytometric analysis of F4/80 macrophages from sham and OVX mice, we assessed mitochondrial reactive oxygen species (mtROS) production by staining cells with 2,7‐Dichlorodi ‐hydrofluorescein diacetate (DCFH‐DA). The mtROS levels of BMMs from OVX mice were higher than those in sham mice (**Figure** **2**a,b). We also analyzed the mitochondrial membrane potential (MMP) of the BMMs. The flow cytometry of tetramethylrhodamine methyl ester (TMRM) showed that the MMP was elevated in BMMs from OVX mice (Figure 2c,d).

Next, we explored whether the expression of specific gene sets was induced under osteoporotic conditions to identify stage‐specific metabolic profiles. We compared the macrophage transcriptomes in sham and OVX mice using RNA‐seq. Principal component analysis (PCA) (Figure S3a, Supporting Information) and hierarchical clustering (HC) (*p* ≤ 0.05) (Figure 2e) of the analyzed genes revealed profound transcriptional differences between macrophages between the sham and OVX groups. We found 1908 dysregulated genes with at least a twofold change (1220 upregulated and 688 downregulated) (Figure 2f) in BMMs treated with OVX compared with Sham‐treated controls. We then performed Gene Ontology (GO) and Kyoto Encyclopedia of Genes and Genomes (KEGG) enrichment analyses based on the RNA‐seq data (Figure 2g; and Figure S3b, Supporting Information). The GO analysis revealed that the enrichments occurring in OVX rats mainly included the regulation of immune effector processes, responses to oxidative stress, mitochondrial organization, and mitochondrial ATP synthesis coupled with electron transport, carbohydrate transport, and glucose transmembrane transport (Figure 2g; and Figure S3c,d, Supporting Information). Metabolism‐related pathways, like HIF‐1 and PI3K‐Akt signaling, were enriched in osteoporosis (Figure S3b, Supporting Information). Furthermore, compared to those of sham mice, BMMs from OVX mice showed enrichment of specific mitochondrial processes, including Complex I, Complex III, reactive oxygen species, glutathione metabolism, carbohydrate metabolism, and the tricarboxylic acid (TCA) cycle (Figure 2h).

### 2.3 Mitochondrial of Macrophages Affects the Osteogenic Differentiation of MSCs

**Figure 3:**
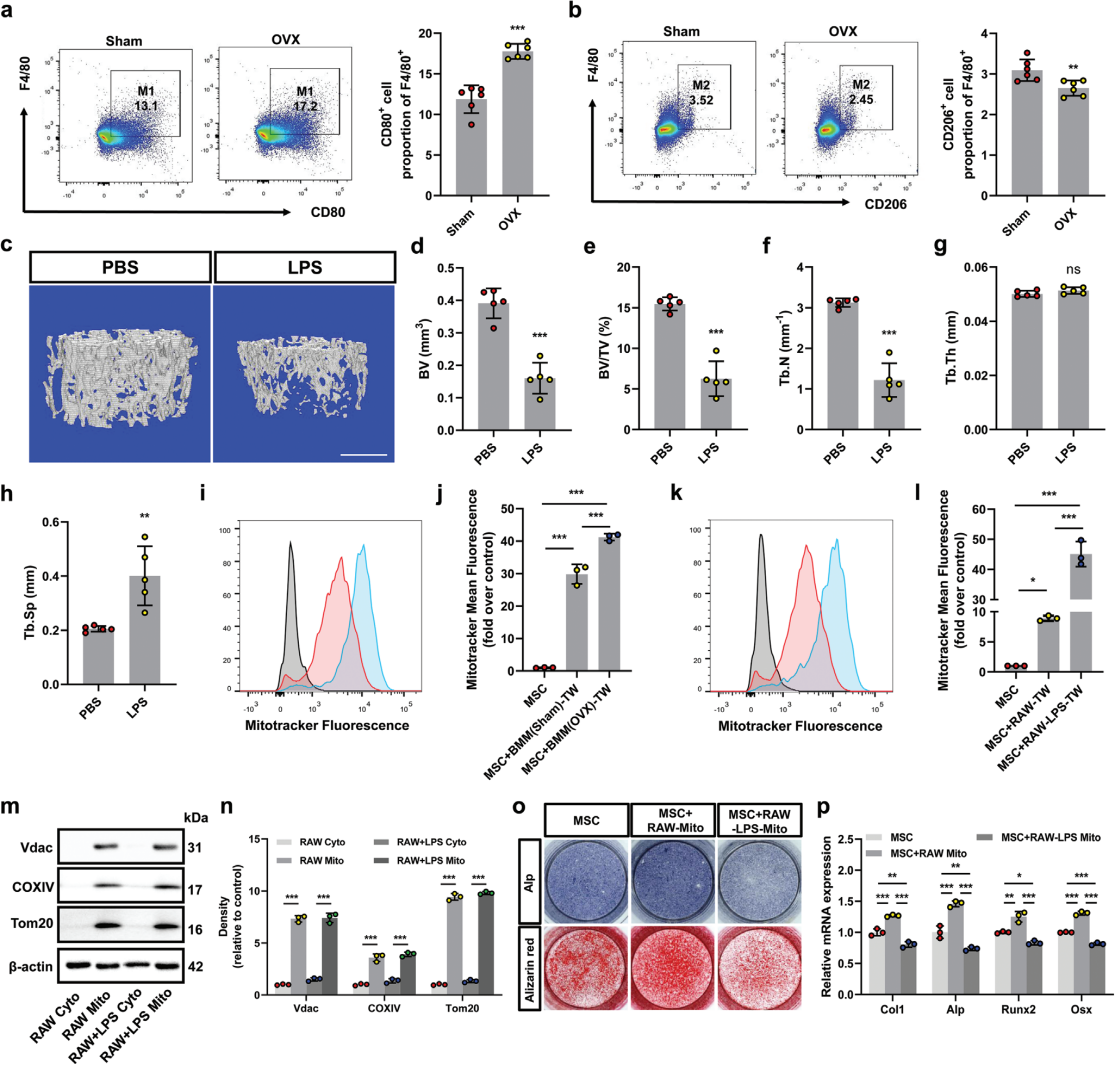
Mitochondrial of macrophages affects the osteogenic differentiation of MSCs. a) Flow cytometry analysis (left) and quantification of CD80^+^F4/80^+^ macrophages in total bone marrow cells (right) (*n* = 6). Two‐tailed Student's *t*‐test. b) Flow cytometry analysis (left) and quantification of CD206^+^F4/80^+^ macrophages in total bone marrow cells (right) (*n* = 6). Two‐tailed Student's *t*‐test. c–h) Representative Micro‐CT 3D structures of distal femurs of PBS and LPS‐treated mice c). Scale bar, 500 µm. And analysis of BV d), BV/TV e), Tb.N f), Tb.Th g), and Tb.Sp h) (*n* = 5). Two‐tailed Student's *t*‐test. i,j) Flow cytometric analysis i) and quantification of mean fluorescence intensity j) of Mitotracker Deep Red‐labeled mitochondrial internalization by MSCs in the presence of sham (red line) or OVX‐treated (blue line) macrophages. Gray line represents untreated MSCs (*n* = 3). One‐way ANOVA with Tukey's post‐test. k,l) Flow cytometric analysis k) and quantification of mean fluorescence intensity l) of Mitotracker Deep Red‐labeled mitochondrial internalization by MSCs in the presence of untreated (red line) or LPS‐treated (blue line) RAW 264.7. Gray line represents untreated MSCs (*n* = 3). One‐way ANOVA with Tukey's post‐test. m,n) Western blotting and quantification of mitochondrial proteins in isolated cytoplasm (Cyto) and mitochondria (Mito) from untreated and LPS‐treated macrophages (*n* = 3). One‐way ANOVA with Tukey's post‐test. o) Representative images of ALP and ARS staining in MSCs incubated with untreated Mito or LPS‐treated Mito, and untreated MSCs (*n* = 3). p) qPCR analysis of *Col1*, *Alp*, *Runx2*, and Osterix (*Osx*) expression under osteogenic conditions (*n* = 3). One‐way ANOVA with Tukey's post‐test. Each error bar represents the mean ± SD of three independent experiments. Differences were considered statistically significant at *p* < 0.05. *p < 0.05, **p < 0.01, and ***p < 0.001.

Macrophages are activated and polarized to M1‐like (usually proinflammatory) or M2‐like (usually anti‐inflammatory) states in response to environmental factors.^[^ ^2^, ^5^ ^]^ We found that in the OVX osteoporosis mice, the percentage of CD80^+^F4/80^+^ M1 phenotype macrophages was significantly increased compared to that of sham mice (**Figure** **3**a). Furthermore, the percentage of CD206^+^F4/80^+^ M2 phenotype macrophages was significantly reduced by OVX, indicating a transition to the M1 phenotype (Figure 3b). To further validate the phenotypic alterations in macrophages, we analyzed proinflammatory gene expression in BMMs using qPCR. Inducible nitric oxide synthase (*Inos*), *IL‐1β*, *TNF‐α*, and *CD80* expression in OVX macrophages was higher than those in sham macrophages (Figure S4a–d, Supporting Information). Furthermore, we found that endotoxin levels in the serum of OVX mice were higher than those in serum of sham mice (Figure S4e, Supporting Information). These results demonstrate that macrophages are in a state of inflammatory activation when osteoporosis occurs.

We used a lipopolysaccharide (LPS)‐induced bone loss model to explore whether higher endotoxin levels activate and polarize macrophages during osteoporosis. A micro‐CT analysis confirmed substantial trabecular bone loss in LPS‐treated mice compared to that in control mice (Figure 3c–h). In LPS‐treated mice, macrophages exhibited phenotypes similar to those in OVX mice, confirming that endotoxin levels affect macrophage polarization and metabolic status (Figure S4f–i, Supporting Information).

Various genetic and chemical stresses trigger increased mitochondrial exchange in various cellular contexts. Inflammatory signaling induced by LPS and TNF‐*α* treatment are strong promoters of mitochondrial exchange.^[^ ^10^, ^22^ ^]^ LPS has been proved to induce mitochondria to transfer to blood stem cells in vivo.^[^ ^23^ ^]^ We also found that mitochondria from macrophages of LPS group transfer more to MSCs (Figure S4j,k, Supporting Information). Similarly, flow cytometric analysis showed that more mitochondria from OVX‐derived macrophages were transferred to MSCs in the OVX group than those in the sham group (Figure 3i,j). More mitochondrial transfer was also found by coculturing RAW 264.7 cells (untreated or treated with LPS) and MSCs (Figure 3k,l).

Next, we investigated whether mitochondria transferred from macrophages to MSCs regulate the osteogenic differentiation of MSCs. Indeed, MSCs that received normally functioning mitochondria showed an enhanced osteogenic phenotype compared with the MSC‐alone group (Figure S5a–e, Supporting Information). In contrast, MSCs receiving l mitochondria (OVX or LPS treatment) showed impaired osteogenic capacity, such as decreased alkaline phosphatase (ALP) levels, decreased alizarin red‐stained mineralized nodules, and decreased osteogenic gene expression (Figure S5f–j, Supporting Information). Then, we clarified how altered osteogenic function is influenced by mitochondrial transfer from macrophages. We extracted intact macrophage mitochondria and analyzed voltage‐dependent anion channel (*Vdac*), cytochrome c oxidase subunit IV isoform 1 (*COXIV*), and translocase of outer mitochondrial membrane 20 (*Tom20*) expression. Western blot analysis confirmed that LPS did not affect the content and expression of mitochondria in macrophages (Figure 3m,n). However, compared with only MSCs and MSCs cultured with normal mitochondria, MSCs cultured with LPS‐treated mitochondria showed lower ALP levels, fewer mineralized nodules, and lower osteogenic gene expression (Figure 3o,p). MSCs have typical stem cell characteristics (Figure S2e, S5k–m, Supporting Information), mitochondria derived from proinflammatory M1 macrophages will not only affect the osteogenic differentiation of MSCs, but also decrease their proliferation and expression of “stemness” markers (Figure S5n–q, Supporting Information). Furthermore, flow cytometry analysis showed that the mitochondria of proinflammatory M1 macrophages would increase the percentage of apoptosis in MSCs s (Figure S5r, Supporting Information).

### 2.4 Macrophages and Derived Mitochondria Affect the Metabolic Status of MSCs

**Figure 4:**
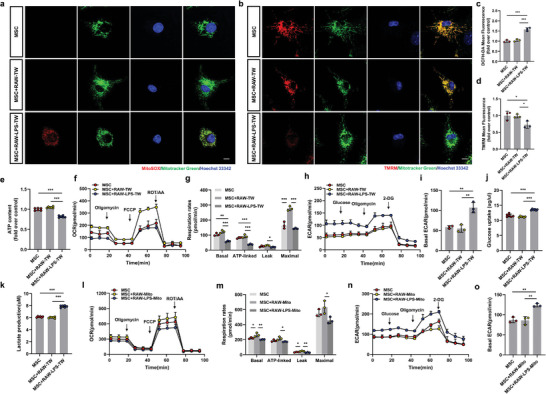
Macrophages and their derived mitochondria affect the metabolic status of MSCs. a,c) Mitochondrial ROS production was analyzed by MitoSOX and DCFH‐DA staining. The mean fluorescence intensity was quantified as a measure of cellular reactive oxygen species production (*n* = 3). Scale bar, 5 µm; one‐way ANOVA with Tukey's post‐test. b,d) Analysis by confocal microscopy and FACS to quantify MMPs. The intensity of TMRM staining reflected MMP (*n* = 3). Scale bar, 5 µm; one‐way ANOVA with Tukey's post‐test. e) Relative ATP levels of MSCs incubated with untreated or LPS‐treated macrophages compared with untreated MSCs (*n* = 6). One‐way ANOVA with Tukey's post‐test. f,g) OCR f), basal respiration, ATP‐linked respiration, proton leak, and maximal respiration g) of the indicated cells (*n* = 3). Two‐way ANOVA with Tukey's post‐test. h,i) ECAR of indicated cells *(n* = 3). One‐way ANOVA with Tukey's post‐test. j,k) Effects of glucose uptake j) and lactate production (k) of MSCs cultured alone or in the presence of untreated or LPS‐treated macrophages (*n* = 6). One‐way ANOVA with Tukey's post‐test. l,m) OCR l), basal respiration, ATP‐linked respiration, proton leak, and maximal respiration m) of MSCs cultured alone or in the presence of untreated Mito or LPS‐treated Mito (*n* = 3). Two‐way ANOVA with Tukey's post‐test. n,o) ECAR of MSCs cultured alone or in the presence of untreated Mito or LPS‐treated Mito (*n* = 3). One‐way ANOVA with Tukey's post‐test. Each error bar represents the mean ± SD of three independent experiments. Differences were considered statistically significant at *p* < 0.05. *p < 0.05, **p < 0.01, and ***p < 0.001.

Energy metabolism in MSCs is closely related to osteoporosis occurrence and progression.^[^ ^2b^ ^]^ We investigated how mitochondrial transport from macrophages to MSCs affects the MSC metabolic state by measuring mtROS and MMP levels in MSCs. Mitochondria from LPS‐treated macrophages led to increased ROS levels in MSCs, as measured by MitoSOX and DCFH‐DA assays (**Figure** **4**a,c). Furthermore, the MMP of MSCs was significantly decreased (Figure 4b,d).

We measured the steady‐state ATP levels in MSCs to explore potential changes in bioenergetics (Figure 4e). Increased ROS production and dysregulated ATP metabolism in MSCs demonstrated impaired mitochondrial function and reduced electron transport chain efficiency. Subsequently, we studied the impact of mitochondrial transfer on MSCs by metabolic profiling using the Seahorse platform. As previously described, we assessed basal respiration, proton leakage, and nonmitochondrial respiration.^[^ ^24^ ^]^ Exposure to untreated macrophages enhanced respiration in MSCs. This transfer‐enhanced respiration was abolished when macrophages were pretreated with LPS (Figure 4f,g). Furthermore, we estimated the cellular glycolytic activity by assessing the extracellular acidification rate (ECAR) in MSCs. An increased ECAR level was observed after MSCs were incubated with LPS‐treated macrophages (Figure 4h,i). This metabolic shift to glycolysis was further supported by elevated glucose uptake (Figure 4j) and increased cellular lactate levels (Figure 4k). Finally, MSCs were incubated with mitochondria from untreated and LPS‐treated macrophages. We observed that LPS‐treated mitochondria promoted glycolysis (Figure 4l,m) and reduced oxygen consumption (Figure 4n,o).

### 2.5 Mitochondrial Transfer Causes Abnormal Succinate Accumulation in MSCs

**Figure 5:**
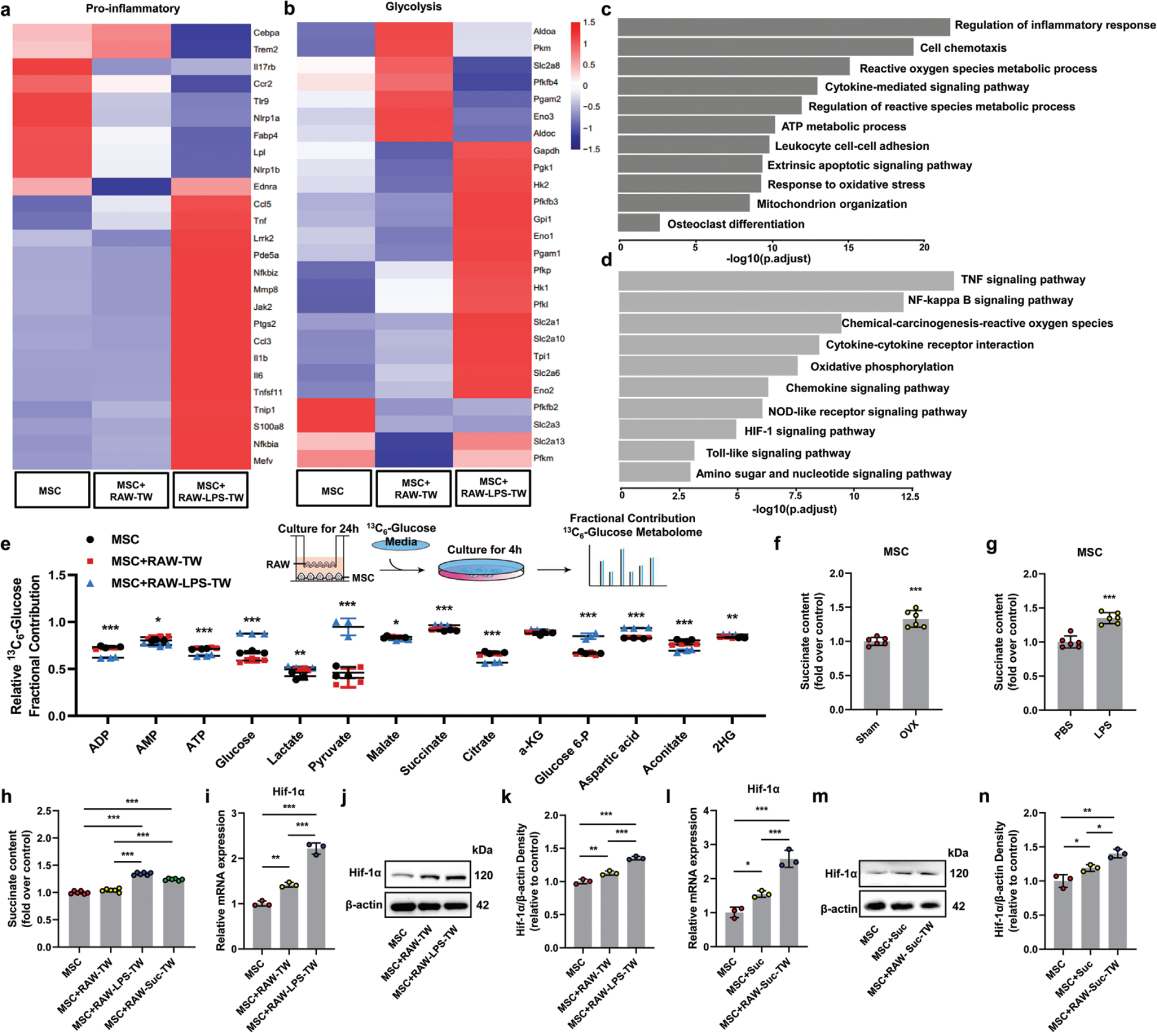
Mitochondrial transfer causes abnormal accumulation of succinate in MSCs. a–d) RNA‐seq analysis of MSCs cultured alone or in the presence of untreated or LPS‐treated macrophages. Pro‐inflammatory genes a), glycolysis genes b), GO analysis c), KEGG analysis d) (*n* = 3). e) Partial contribution of ^13^C_6_‐glucose to the selection of relevant metabolites (*n* = 3); one‐way ANOVA with Tukey's post‐test. f,g) Relative levels of succinate in MSCs of Sham, OVX, PBS, and LPS‐treated mice (*n* = 6). Two‐tailed Student's *t*‐test. h) Succinate content in MSCs cultured alone or in the presence of untreated or LPS‐treated or succinate‐treated macrophages (*n* = 3). One‐way ANOVA with Tukey's post‐test. i–k) qPCR analysis i) and western blotting j,k) to determine the levels of Hif‐1*α* in MSCs incubated with untreated or LPS‐treated macrophages compared to untreated MSCs (*n* = 3). One‐way ANOVA with Tukey's post‐test. l–n) qPCR analysis l) and western blotting m,n) to determine the levels of Hif‐1*α* in MSCs incubated with succinate or succinate‐treated macrophages compared to untreated MSCs (*n* = 3). One‐way ANOVA with Tukey's post‐test. Each error bar represents the mean ± SD of three independent experiments. Differences were considered statistically significant at *p* < 0.05. *p < 0.05, **p < 0.01, and ***p < 0.001.

To investigate the molecular basis of metabolic reprogramming of MSCs by mitochondrial transfer, we analyzed the MSC transcriptome. We found that proinflammatory gene expression (including *IL‐6* and *IL‐1β*) were increased in the M1‐like macrophage group compared with that in the untreated macrophage and MSC groups (**Figure** **5**a). Interestingly, glycolytic genes were also upregulated (Figure 5b), consistent with the increased ECAR (Figure 4h,i). We then performed GO and KEGG enrichment analyses on the RNA‐seq data. GO analysis showed that the MSCs in the oxidatively damaged mitochondrial transfer group showed changes in ROS metabolic process, ATP metabolic process, and mitochondrial organization (Figure 5c). Furthermore, we found that the genes involved in the regulation of inflammatory responses related to inflammatory activation and osteoclast differentiation in bone remodeling‐related systems also changed (Figure 5c). MSCs in the oxidatively damaged mitochondrial transfer group were enriched in inflammatory and metabolism‐related pathways such as the TNF‐*α* signaling pathway, NF‐*κ*B signaling pathway, oxidative phosphorylation, Hif‐1*α* signaling pathway, and Toll‐like signaling pathway (Figure 5d).

Subsequently, we labeled MSCs with ^13^C_6_‐glucose and used mass spectrometry to trace the fate of glucose isotopes (Figure 5e). We found that the flux of glucose metabolism, was significantly altered in MSCs after oxidatively damaged mitochondrial transfer. Furthermore, in the M1‐like macrophage group, succinate, a TCA cycle intermediate, was elevated (Figure S6a, Supporting Information). We observed succinate accumulation in MSCs in the osteoporosis‐induced mice compared to that in mice in the sham and PBS groups (Figure 5f,g). In BMMs and serum of osteoporosis model mice, the succinate content also increased (Figure S6b–e, Supporting Information). Interestingly, macrophages pretreated with succinate (5 mm) also influenced the succinate levels in MSCs (Figure 5h). This abnormal succinate accumulation in MSCs of the oxidatively damaged mitochondrial transfer group may be related to the decreased activity of oxidases, such as succinate dehydrogenase (SDH) (Figure S6f, Supporting Information).

Hif‐1*α* plays a key role in glucose metabolism and bone resorption, and its activation is regulated by increased succinate levels.^[^ ^21^, ^25^ ^]^ Moreover, a sustained increase in succinate induces proinflammatory cytokine expression (such as IL‐1*β*) via Hif‐1*α*.^[^ ^21^, ^26^ ^]^ Accordingly, *Hif‐1α* gene and protein expression in MSCs in the presence of LPS‐treated macrophages were higher than those in the MSCs and untreated groups (Figure 5i–k). In addition, after pretreating macrophages with succinate, MSCs showed Hif‐1*α* activation (Figure 5l–n), upregulated glycolytic genes such as hexokinase 2 (*Hk2*) and solute carrier family 2 member 1 (*Slc2a1*), and upregulated pro‐inflammatory genes such as *IL‐1β* and *TNF‐α* compared to those in the blank control group (Figure S6g–p, Supporting Information). Moreover, succinate oxidation inhibitor dimethyl malonate (DMM) can significantly affect the expression of proinflammatory factors and osteogenic indicators in MSCs induced by succinate (Figure S6q, r, Supporting Information). These data suggest that succinate may directly regulate Hif‐1*α* signaling and proinflammatory cytokine release, thereby affecting the osteogenic differentiation of MSCs.

### 2.6 Mitochondrial Transfer Maintains Bone Health In Vivo

**Figure 6:**
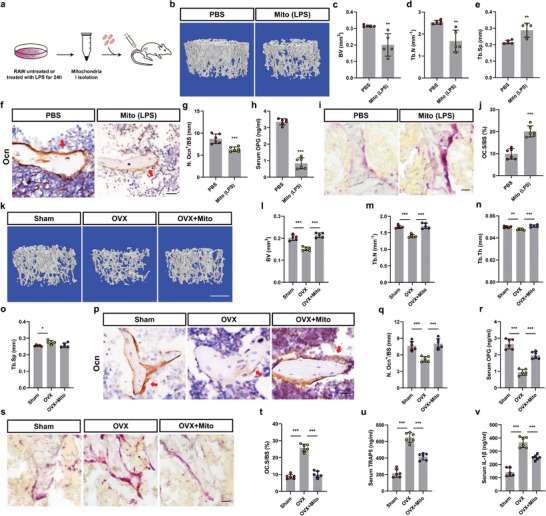
Mitochondrial transfer maintains bone health in vivo. a) Schematic showing mitochondrial application and treatment. b–e) Mice received tail vein injections of LPS‐treated Mito (100 µg dissolved in 100 µL PBS) or PBS twice a week for 3 weeks. Bone phenotypes were then analyzed b). Scale bar, 500 µm. BV c), Tb.N d), and Tb.Sp e) analysis (*n* = 5). Two‐tailed Student's *t*‐test. f,g) Representative images of osteocalcin (Ocn) staining f) and quantification of number of Ocn+ cells (red arrows) in femurs g). Scale bars, 25 mm (*n* = 6). Two‐tailed Student's *t*‐test. h) Serum Osteoprotegerin (OPG) level in PBS and mito (LPS) mice (*n* = 6). two‐tailed Student's *t*‐test. i,j) Representative images of representative TRAP staining of the distal femur in PBS and mito (LPS) mice i). Scale bar, 100 µm. Quantification of the area of osteoclasts on the surface of trabecular bone (OC.S/BS) j) (*n* = 6). Two‐tailed Student's *t*‐test. k–o) Representative Mico‐CT images of femurs from Sham, OVX and OVX+Mito mice k). Scale bar, 500 µm. BV l), Tb.N m), Tb.Th n), and Tb.Sp o) analysis (*n* = 5). One‐way ANOVA with Tukey's post‐test. p,q) Representative images of osteocalcin (Ocn) staining p) and quantification of number of Ocn+ cells (red arrows) in femurs q) in Sham, OVX, and OVX+Mito mice. Scale bars, 25 mm (*n* = 6). One‐way ANOVA with Tukey's post‐test. r) Serum OPG level in Sham, OVX, and OVX+Mito mice (*n* = 6). s,t) Representative images of representative TRAP staining of the distal femur in Sham, OVX, and OVX+Mito mice s). Scale bar, 100 µm. The area of osteoclasts (OC.S/BS) on the surface of trabecular bone was quantified t) (*n* = 6). one‐way ANOVA with Tukey's post‐test. u,v) Serum TRAP5 u) and IL‐1*β* v) levels in Sham, OVX, and OVX+Mito mice (*n* = 6). One‐way ANOVA with Tukey's post‐test. Each error bar represents the mean ± SD of three independent experiments. Differences were considered statistically significant at *p* < 0.05. *p < 0.05, **p < 0.01, and ***p < 0.001.

To examine the effect of mitochondria in M1‐like macrophages on skeletal health, we administered PBS and LPS‐treated macrophage mitochondria [mito (LPS)] via the tail vein of 8‐week‐old mice twice a week for 4 weeks and analyzed their skeletal phenotype (**Figure** **6**a). Consistent with the Von Kossa staining (Figure S7a, Supporting Information), a micro‐CT analysis revealed lower bone volume, trabecular volume, trabecular number, and higher trabecular separation in the mito (LPS‐treated) group than in the PBS group (Figure 6b–e; and Figure S7b, Supporting Information). Meanwhile, compared with the PBS group, the number of osteocalcin^+^ osteoblasts and the serum OPG concentration of the mice were decreased in the mito (LPS) group (Figure 6f–h).

We measured osteoclast bone resorption in mito (LPS) mice versus control mice and found that mito (LPS)‐treated mice had more tartrate‐resistant acid phosphatase (TRAP)+ osteoclasts on the trabecular bone surface than in PBS‐treated controls (Figure 6i,j). In addition, serum TRAP5 and IL‐1*β* concentrations were also higher in the mito (LPS) group than in the PBS group (Figure S7c,d, Supporting Information). Taken together, these results suggest that mitochondria in M1‐like macrophages disrupt skeletal homeostasis.

Finally, we tested the effects of mitochondrial transfer on osteoporosis in vivo. We treated OVX mice by injecting mitochondria twice a week via the tail vein. Von Kossa staining and micro‐CT analysis showed that the bone volume, trabecular volume, trabecular number, and trabecular thickness in OVX mice were significantly lower than those in sham group, while all these down‐regulated parameters induced by OVX were reversed after mitochondria treatment (Figure 6k–o; and Figure S7e, Supporting Information). Immunohistological analysis and detection of serum markers showed that osteocalcin^+^ osteoblasts and OPG concentrations in OVX+mito were also increased compared with OVX group (Figure 6p–r). Furthermore, the TRAP+ osteoclasts were significantly reduced in OVX+mito‐treated mice compared to those in OVX‐treated mice (Figure 6s,t). Compared with the sham group, the serum TRAP5 and IL‐1*β* concentrations in the OVX group were increased, and there was a certain decrease in the OVX+mito group (Figure 6u,v).

## 3 Discussion

**Figure 7:**
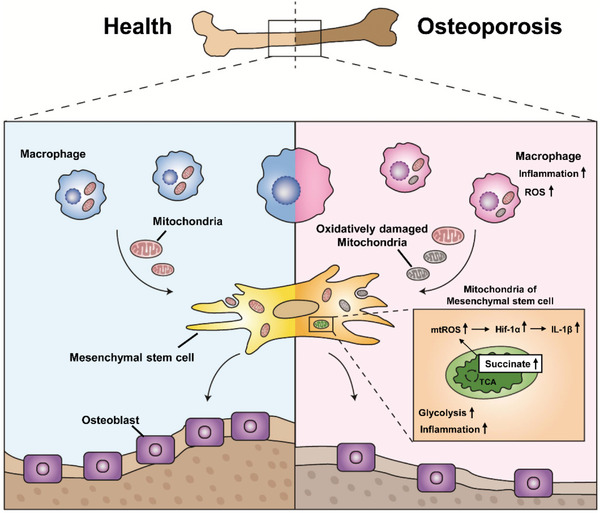
Schematic showing the mode of action of intercellular mitochondrial transfer of macrophages and MSCs in the bone environment to affect bone metabolism. Our findings reveal that mitochondrial transfer may have emerged as a new mode of regulating cellular crosstalk in the bone environment. When macrophages are in an osteoporotic state, they release mitochondria with oxidative damage, triggering elevated ROS, and mitochondrial dysfunction in MSCs. Metabolic dysfunction in MSCs leads to intracellular succinate accumulation, which promotes elevated levels of inflammation, which in turn affects osteogenic differentiation.

Our results demonstrated that macrophages in the bone marrow environment regulate the bioenergetic status and osteogenic differentiation of MSCs by transferring their mitochondria into MSCs. Under osteoporotic conditions, macrophages undergo oxidative abnormalities and transition into proinflammatory M1‐like cells. This process leads to increased mitochondrial transfer from macrophages to MSCs. Moreover, increased mitochondrial transfer from M1 macrophages MSCs induces cellular damage and metabolic reprogramming. We also demonstrated that metabolic intermediates of the TCA cycle, such as succinate, increased in osteoporotic mice. Succinate regulates inflammation and is involved in multiple biological processes as a key signal. Succinate accumulation in MSCs promotes the increased expression of proinflammatory genes, including *IL‐1β*, by inducing elevated ROS levels and Hif‐1*α* activation, which in turn affects the osteogenic differentiation of MSCs (**Figure** **7**).

Recently, the relationship between macrophage polarization and changes in the bone marrow environment have received extensive attention.^[^ ^2^, ^27^ ^]^ Under osteoporotic conditions, macrophages in the bone marrow environment exhibit altered mitochondrial functions, such as elevated ROS, increased mitochondrial membrane potential (MMP), and a shift to glycolysis. Moreover, the development of various inflammatory states is regulated by endotoxin levels.^[^ ^2^, ^28^ ^]^ We observed elevated endotoxin levels in osteoporotic OVX mice, which further confirms that osteoporosis is a chronic inflammatory disease. To simulate impaired mitochondrial function in vitro, macrophages were treated with LPS. Changes in the mitochondrial function and the metabolic state of macrophages after LPS treatment were achieved by inhibiting complex II and III and remission state III respiration.^[^ ^21^, ^29^ ^]^

Mitochondria released from multiple cell types have been studied using in vitro and in vivo methods to validate cell‐to‐cell transfer.^[^ ^9^, ^30^ ^]^ However, the main focus of these studies is that damaged or cancer cells obtain mitochondria from donor cells (most likely MSCs) to rescue the metabolic and proliferative needs of the damaged cells.^[^ ^20^, ^31^ ^]^ There are far fewer reports on the transfer of mitochondria to MSCs. Only a few reports indicate that this phenomenon may be involved in the antiapoptotic and prodifferentiation functions of activated stem cells.^[^ ^32^ ^]^ Levoux et al. demonstrated that platelets enhance the therapeutic effect of MSCs on wound healing, whereas platelets with dysfunctional mitochondrial affect the therapeutic capacity of MSCs.^[^ ^9a^ ^]^ The donor cell type and functional status may have different effects on recipient cells after mitochondrial transfer.^[^ ^31^, ^33^ ^]^ A similar phenomenon was observed in our study; when infused with mitochondria from M1‐like macrophages (LPS‐treated), mice developed osteoporosis‐like symptoms, whereas infusing normal mitochondria ameliorated the bone loss symptoms after OVX surgery.

Substantial evidence suggests that cell‐to‐cell mitochondrial transfer occurs more frequently in organisms than previously thought.^[^ ^9^, ^20^, ^32^ ^]^ Mitochondrial transfer between cells regulates cellular function and the energy metabolism state of recipient cells through mitochondrial integration.^[^ ^9^, ^11^ ^]^ In our experiments, we observed that when M1‐like macrophage mitochondrial transfer to MSCs increased, mitochondrial abnormalities, such as increased ROS and decreased MMP were induced. Simultaneously, we found that oxygen consumption in MSCs decreased and glycolysis increased during this process. This evidence suggests that increased transfer of abnormal mitochondria alters the metabolic state of MSCs, which in turn leads to osteogenic differentiation disorders.

Subsequently, we verified how mitochondria from M1‐like macrophages affect TCA cycle metabolism in recipient MSCs. Several intermediates of the TCA cycle, including succinic acid, were upregulated. Succinate is an important metabolite in host and microbial processes and is involved in various pathophysiological processes in vivo.^[^ ^34^ ^]^ Circulating succinate levels are elevated in metabolic and inflammation‐related diseases, including ischemic heart disease,^[^ ^35^ ^]^ obesity,^[^ ^36^ ^]^ and type 2 diabetes.^[^ ^3^, ^36^ ^]^ Guo et al. concluded that hyperglycemia induces succinate accumulation in bone marrow stromal cells, which in turn stimulates osteoclastogenesis.^[^ ^3^ ^]^ The development of metabolic disorders in skeletal disorders is controlled by succinate, but the mechanism remains unknown. Notably, abnormal ROS elevation during ischemia‐reperfusion injury is controlled by succinate accumulation, and ROS levels affect the severity of tissue damage.^[^ ^37^ ^]^

In addition to inducing ROS production, high succinate levels directly inhibit the prolyl hydroxylase domain (PHD) to regulate Hif‐1*α* stability. Succinate accumulation induces the transcription of many inflammatory genes, including those encoding IL‐1*β*, through Hif‐1*α*, which plays an important role in glycolytic reprogramming.^[^ ^21b^ ^]^ However, local transplantation of human MSC‐EVs also induces Hif‐1*α* expression, and Hif‐1*α*‐mediated *Vegfa* expression is a key regulator that promotes angiogenesis and accelerates fracture healing.^[^ ^38^ ^]^ This mechanism is consistent with the results of our OVX recovery experiments. We observed that Hif‐1*α* increased when macrophages and MSCs were cocultured, but MSCs showed enhanced osteogenic differentiation ability. We hypothesized that MSC differentiation ability may be affected by succinate‐regulated inflammatory genes. Succinate levels did not increase in MSCs after normal mitochondrial transfer. Therefore, inflammatory activation is regulated by intracellular succinate levels in MSCs after mitochondrial transfer and is critical for MSC metabolism and differentiation.

This study focused on mitochondrial delivery from macrophages to MSCs for two reasons. 1) Our experiments verified whether the bone marrow environment in osteoporosis is in an inflammatory state and macrophages respond faster and more directly. 2) MSCs, a stem cell population that provides osteoblasts in the bone marrow environment, have high research and application potential to study the effects of other factors on their cell status. In future experiments, we will examine mitochondrial transfer from MSCs to macrophages and their regulation in more detail.

Our research has several noteworthy limitations. Our study focused on the mitochondrial transfer of proinflammatory M1 macrophages to MSCs, and did not reveal the mitochondrial transfer to osteoclasts. Therefore, our further work will aim to understand the mitochondrial transfer of proinflammatory M1 macrophages to cells in a variety of bone microenvironments and how they affect their physiological functions. Second, we confirmed the effect of pro‐inflammatory M1 macrophages on MSCs by various means in vivo and in vitro. However, how to interfere with proinflammatory M1 mitochondrial transfer and achieve targeted treatment for osteoporosis remains to be explored. Use the confirmed form of mitochondrial transfer to inhibit the formation of endocytosis, exocytosis and TNT, and determine whether blocking mitochondrial transfer can reverse metabolic diseases such as osteoporosis.

## 4 Conclusion

Our findings reveal a new pattern of cellular crosstalk in the bone marrow environment, with macrophages regulating bone metabolism crosstalk by transferring their mitochondria into MSCs. This study suggests that mitochondrial transfer may be a novel way to regulate homeostasis by enabling immune cells to regulate their local tissue environment. Targeting mitochondrial transfer to prevent bone loss provides a new approach to the treatment and prevention of osteoporosis and proposes additional strategy to improve metabolism‐related diseases.

## 5 Experimental Section

### Mice

Female C57BL/6 mice were obtained from the Animal Center of Wuhan University. All experimental procedures were approved by Wuhan University and were performed according to laboratory animal care and use guidelines. The study protocol was approved by the Ethics Committee for Animal Use of the Institute of Biomedical Sciences (Protocol number 69/2017).

For the LPS (Solarbio) induced calcium osteolysis mouse model, female C57BL/6 mice (8 weeks old) were randomly assigned to three groups: PBS group (*n* = 10), LPS group (5 mg kg^−1^ LPS, *n* = 10), and mito (LPS) group (100 µg LPS‐Mito, *n* = 10). LPS (5 mg kg^−1^) was injected intraperitoneally once per week for 3 weeks. Mito (LPS) (100 µg dissolved in 100 µL PBS) was injected through the tail vein twice weekly for 3 weeks.

Ovariectomy was performed at 8 weeks of age, as described previously.^[^ ^39^ ^]^ For the postmenopausal osteoporosis model, the mice were divided into three groups: sham mice (sham, *n* = 10), OVX mice (OVX, *n* = 12), and Mito mice (OVX + Mito, *n* = 10). RAW Mito (100 µg dissolved in 100 µL PBS) was injected through the tail vein twice a week starting 1 week after surgery for 6 weeks. Blood samples were centrifuged at 1000 × g for 15 min to harvest the serum, which was stored at −80 °C for later use. Femurs were also collected.

For the BMMs transplantation experiment, mice received 8 Gy irradiation from a cesium‐137 irradiator. Mitochondria from BMMs overexpressing Tom20‐GFP were intravenously injected into irradiated mice. At 24 h after irradiation, 1 × 10^6^ BMM Tom20‐GFP cells were transplanted via a tail vein injection. The mice were analyzed 24 h after transplantation.

For serum markers of bone formation and bone absorption, mouse serum was collected for ELISA (Bioswamp) analysis of OPG and TRAP5 levels. All procedures are carried out in accordance with the manufacturer's instructions.

### BMM and MSC Isolation and Culture

BMMs were extracted from the leg bones, differentiated in DMEM (Gibco) containing 10% fetal bovine serum (FBS, Gibco), 1% penicillin streptomycin (Gibco), and macrophage colony‐stimulating factor (M‐CSF) (50 ng mL^−1^) (Proteintech) for 72 h. Unless otherwise stated, 5.0 × 10^5^ BMM mL^−1^ were used for in vitro experiments.

MSCs were originally isolated from the tibias and femurs of mice. The bone marrow was washed with *α*‐Dulbecco's medium (*α*‐MEM; Gibco). The adherent cell population was left behind in standard *α*‐MEM containing 20% FBS. The cells were further purified by passage, and cells from passage 3 were used for subsequent experiments. The cells were grown in a humidified 5% CO_2_ atmosphere at 37 °C.

### Assessment of Mitochondrial Transfer From Macrophages to MSCs

To evaluate mitochondrial transfer, MSCs and macrophages were labeled with MitoTracker Green (100 nm, Invitrogen) and MitoTracker Deep Red (100 nm, Invitrogen), respectively. The intercellular mitochondrial transfer of MSCs (green) and macrophages (red) was photographed at 0, 3, 6, and 24 h after coculture using an LSM800 confocal microscope (Zeiss), and videos of mitochondrial transfer within 3 h of coculture were captured.

Furthermore, we assessed mitochondrial transfer in Transwell plates containing 0.4 µm pore polycarbonate membranes (Millipore). MSCs (green) and macrophages (red) were labeled with mitochondrial dyes, cultured in a 24‐well plate containing Transwell for 24 h, and then photographed using a confocal microscope.

Macrophage‐derived mitochondria in MSCs were further detected by LSR Fortessa X20 flow cytometer (BD Biosciences). MSCs were seeded into 12‐well plates at a density of 16 × 10^4^ per well for 24 h. RAW and BMM were incubated with MitoTracker Deep Red (100 nm, Invitrogen) for 30 min. They were then washed 3 times with PBS and seeded into 12‐well plates and cultured for an additional 24 h. After incubation, cells in wells were harvested and incubated with F4/80‐PE‐Cy7 (Biolegend; 1:400) antibody for 30 min at 4 °C. Flow cytometric collection of MitoTracker Deep Red in F4/80^−^ cells. Data from flow cytometry were representative of receiving macrophage‐derived mitochondria in MSCs. The mean fluorescence intensity of received mitochondria in MSCs was analyzed using FlowJo software.

Finally, after 24 h of treatment with 50 µm dynasore, a dynein‐dependent clathrin‐mediated endocytosis inhibitor (MedChemExpress), analysis by flow cytometry and cytometry kit‐8 (CCK8) viability assay Mitochondrial transfer and MSC viability.

### Flow Cytometry

As previously described,^[^ ^40^ ^]^ MSCs exhibited the following phenotypes: CD29^+^ (BioLegend; 1:200), stem cell antigen 1 (Sca‐1)^+^ (Invitrogen; 1:200), CD73^+^ (BioLegend; 1:200), CD44^+^ (BioLegend; 1:100), CD34^−^ (BioLegend; 1:100), and CD45^−^ (BioLegend; 1:200). BMMs were incubated with F4/80‐PE‐Cy7 (Biolegend; 1:400), CD206‐FITC (BioLegend; 1:200), and CD80‐PE‐Cy5 (BioLegend; 1:200) antibodies at 4 °C for 30 min. Then, the ratios of CD206/F4/80 and CD80/F4/80 cells were analyzed. Labeled cells were collected and analyzed using a FACScan flow cytometer (Becton Dickinson). Data were analyzed using FlowJo software.

### Osteogenic Differentiation Assay

For osteoblast differentiation, MSCs were cultured in DMEM containing 10% FBS, 100 nm dexamethasone (Sigma), 50 µg mL^−1^ ascorbic acid (Sigma), and 5 mm *β*‐glycerophosphate (Sigma). After culturing for 7 days, cells were stained using a BCIP/NBT alkaline phosphatase color reagent kit (Beyotime) to evaluate alkaline phosphatase content. After incubation for 14 days, cells were stained with 0.2% alizarin red (pH 4.2, Sigma‐Aldrich) to evaluate the degree of mineralization.

### Cell Proliferation and Apoptosis Assay

The proliferation of MSCs was evaluated using Cell Count Kit‐8 (MCE). After the cells were treated with CCK‐8 reagent, they were incubated for 2 h, and the optical density (OD) value at 450 nm was measured with a spectrophotometer.

MSCs apoptosis was measured with FITC labeled Annexin‐V. Cell necrosis was measured with propidium iodide (PI) (Solarbio). After exposure to various experimental conditions, cells were trypsinized and labeled with fluorescent dye at 37 °C. The FACS scanner was used for cell fluorescence analysis.

### Transmission Electron Microscopy

MSCs were seeded at a density of 16 × 10^4^ cells mL^−1^ in 12‐well plates and cocultured with RAW 264.7 cells (mouse mononuclear macrophage leukemia cells) for 24 h. The cells were fixed with 3% glutaraldehyde for 5 min at room temperature, washed three times with PBS, and post‐fixed with 1% osmium tetroxide. The fixed cells were dehydrated in an ascending ethanol series (70%, 90%, and 100%) and infiltrated and embedded in epoxy resin.^[^ ^11b^ ^]^ After the samples were sliced (70 nm) with a diamond knife, the sections were picked using a copper grid (100 mesh). After staining with uranyl acetate and lead citrate, the samples were observed using a JEM‐1400 transmission electron microscope (JEOL) at an acceleration voltage of 120 kV.

### Mitochondrial Isolation and Quantification

The method for isolating mitochondria from the cells was optimized according to the manufacturer's protocol (Solarbio). Conditioned cells were washed with PBS and collected. Ice‐cold lysis buffer was added to resuspend the cells, which were lysed with a tissue homogenizer in an ice bath at 0 °C. Mitochondria were obtained after gradient centrifugation. The mitochondrial protein concentration was determined using the BCA protein quantification Kit.

### Real‐Time PCR Assays

Total RNA from BMM and MSC were isolated using TRIzol reagent (Takara). cDNA was prepared by reverse transcription of 1 µg total RNA using HiScript II Q RT SuperMix (Vazyme). Amplification reactions were performed using ChamQ SYBR qPCR master mix (Vazyme) and random primers. The 2^−ΔΔCt^ method was used to quantify relative expression, which was normalized to *β*‐actin levels.

### Western Blot

Total intracellular protein was extracted using precooled RIPA lysis buffer. Protein concentrations were detected using a BCA protein assay kit (Beyotime). Samples with equal protein concentrations were separated by 10% SDS‐PAGE. The proteins were transferred to PVDF membranes (Roche). The membranes were blocked with skim milk for 1 h at room temperature and incubated with primary antibody overnight at 4 °C. Membranes were incubated with appropriate HRP‐conjugated secondary antibody (ABclonal; 1:10 000) for 1 h at room temperature. Signals were detected using enhanced chemiluminescence reagent (ECL, Advansta). The following primary antibodies were used for Western blot analysis at the indicated dilutions: anti‐Mfn2 (Proteintech, 1:1000), anti‐Vdac (ABclonal, 1:1000), anti‐COXIV (ABclonal, 1:1000), anti‐Tom20 (ABclonal, 1:1000), anti‐Hif‐1*α* (ABclonal, 1:1000), and anti‐*β*‐actin (ABclonal, 1:1000).

### Endotoxin Measurements and Inflammation Analysis

Serum, BMM, and MSC endotoxin levels were determined using an LAL Chromogenic Endotoxin Quantitation Kit (Pierce). The serum was diluted 1:50 under pyrogen‐free conditions and inactivated at 70 °C for 15 min. Limulus reagent was added and incubated at 37 °C for 8 min. The cells were incubated for 6 min after adding developer. After adding the stop reaction solution, 100 µL sample was pipetted into a pyrogen‐free 96‐well plate and use a microplate reader (Molecular Devices) to measure the absorbance at 405 nm.

In order to analyze inflammation, serum was collected from mice and the concentration of IL‐1*β* in serum was determined by ELISA kit (Bioswamp). All procedures are carried out in accordance with the manufacturer's instructions.

### Confocal and FACS Determination of ROS

MSCs were seeded into 24‐well plates with cell slides at a density of 4 × 10^4^ per well and divided into control, RAW‐Transwell and RAW+LPS‐Transwell groups. Cells were cultured in DMEM for 24 h. Cells were mixed with 3 µm MitoSOX Red (Invitrogen) and 100 nm MitoTracker Green in PBS and incubated at 37 °C for 30 min. The cell slides were transferred into confocal dishes and the mitochondrial ROS of the cells were analyzed by confocal microscopy.

In addition, MSCs were seeded into 12‐well plates at a density of 16 × 10^4^ per well and divided into control, RAW‐Transwell and RAW+LPS‐Transwell groups. After cells were cultured in DMEM for 24 h, cells were mixed with 10 µm DCFH‐DA (Beyotime) and incubated at 37 °C for 20 minand analyzed by flow cytometry (Becton Dickinson) for mean fluorescence intensity. Data were analyzed using FlowJo software.

### Confocal and FACS Determination of Tetramethylrhodamine Methyl Ester

MSCs were seeded into 24‐well plates with cell slides at a density of 4 × 10^4^ per well and divided into control, RAW‐Transwell and RAW+LPS‐Transwell groups. Cells were cultured in DMEM for 24 h. Cells were incubated in TMRM (Invitrogen) (100 nm) for 20 min at 37 °C. Cells were imaged using a confocal microscope (Zeiss, Jena, Germany).

For flow cytometry assays, the cells were trypsinized into single cell suspensions and resuspended in PBS. The TMRM intensity in different groups was measured using a flow cytometer and the data were analyzed using FlowJo software.

### Metabolic Analysis

Energy metabolism analysis was performed using an XF24 extracellular flux analyzer (Seahorse Bioscience). Briefly, cells were seeded into XF24 cell culture plates (Seahorse Bioscience) at 8 × 10^4^ cells per well after treatment as described earlier. The plate containing the syringe ports and probes and the utility plate containing calibrator solution (1 mL per well) were placed together in a CO_2_‐free incubator at 37 °C overnight. The next day, the original medium was replaced with XF assay buffer (XF basic medium supplemented with 25 mm glucose, 1 mm pyruvate, 2 mm L‐glutamine, and adjusted to pH 7.4). The cell culture plate was placed in a CO_2_‐free incubator for at least 0.5 h. Inhibitors [1 µm oligomycin, 1 µm carbonyl cyanide‐4‐(trifluoromethoxy)phenylhydrazone (FCCP), 1 µm rotenone, 10 mm glucose, and 50 mm 2‐Deoxy‐*D*‐glucose (2DG]) were added to the port of the syringe plate. The plate was run using the utility plate for calibration. After completion, the utility plate was replaced with a cell culture plate for the run. Data were analyzed using Seahorse Wave software 2.6.1. The data were normalized using the protein content.

### RNA‐Seq and Data Analysis

Total RNA was extracted from BMM and MSC cells using the TRIzol reagent (Takara). Purified poly(A) mRNA was isolated from 1 mg total RNA using a Poly(A) mRNA Magnetic Isolation Module (Thermo Scientific). Then, RNA‐seq libraries were prepared using NEBNext Super Directional RNA Library Preparation Kits (New England Biolabs). Sequencing was performed on an Illumina NovaSeq 6000 platform with 150‐bp paired‐end reads. RAW sequencing data were processed by Trim_Galore (v.0.6.7) for removing adapters and low‐quality base pairs. The reads were mapped to the mouse reference genome (GRCm38) using HISAT2 software (v.2.1.0). The number of reads mapped to each Ensembl gene was counted using featureCounts (v.2.0.1). Sequence count data normalization and differential expression analysis was performed using R/Bioconductor package DESeq2 (v1.32.0). RNA‐seq data have been deposited at GEO (GSE198806) and are publicly available as of the date of publication.

### Micro‐CT Analysis

Mouse femurs were fixed with 4% paraformaldehyde for 24 h at 4 °C and scanned using a high‐resolution micro‐computed tomography scanner (SkyScan). The scanner was set at 80 kV, 100 mA, and 6 mm per pixel resolution. The region of interest (ROI) was analyzed from 0.1 mm below the growth plate to 5% of the femur length. A 3D analysis of the following morphological parameters was performed using CTAn (Brucker): bone volume (BV), trabecular bone volume per tissue volume (BV/TV), trabecular number (Tb.N), trabecular thickness (Tb.Th), and trabecular separation (Tb.Sp).

### Histochemistry and Immunohistochemical Analysis

For histochemistry, the femur was fixed with 4% paraformaldehyde at 4 °C for 24 h, decalcified with 10% EDTA (pH 7.4) for 14–21 days, and then embedded in paraffin. Preparation with a slicer (Leica) 5 µm sections were stained with von Kossa and TRAP (Sigma). Bone histomorphology parameters 69 and ImageJ were used for image analysis.

For immunohistochemical staining, use 0.05% trypsin to digest the sample at 37 °C for 15 min. The sections were incubated with anti‐Osteocalcin antibody (1:200; SantaCruz) at 4 °C overnight. Then use DAB substrate kit (MXB biotechnology) for color reaction to detect antibody binding.

### ^13^C_6_‐Glucose LC‐MS Metabolomics

MSCs were seeded into 6‐well plates at a density of 40 × 10^4^ per well and were divided into control, RAW‐Transwell, and RAW+LPS‐Transwell groups. The cells were cultured in DMEM for 24 h. The media was then replaced with homogeneously labeled DMEM containing 25 mm **^1^** ^3^C**_6_**‐glucose (Sigma) and incubated for 4 h. The cells were gently washed three times with ice‐cold PBS. Then, 1 mL prechilled 80% methanol was added to each well, and the cells were lysed at –80 °C for 2 h. The lysate was collected and centrifuged at 14 000 × g for 20 min at 4 °C. The protein concentrations in the extracts were determined using a BCA protein quantification Kit. The supernatant was dried using a Speedvac instrument. Metabolite measurements were performed using the Tsinghua University Metabolomics platform. **^1^** ^3^C enrichment in glucose was determined using LC‐MS.^[^ ^21b^ ^]^ The expected retention times and accurate masses were measured using TraceFinder 4.1 (Thermo Scientific) and target metabolites were quantified using the area under the curve. Cell numbers and sample protein concentrations were used for normalization. The relative metabolite levels were calculated by summing the values for all isotopologs of a given metabolite. The ^13^C carbon relative to the total carbon was calculated for each metabolite.

### Colorimetric Determination of Succinate

We used a succinate (succinic acid) assay kit (BioVision Inc.) according to the manufacturer's manual. Succinate levels were detected in serum, BMM, and MSC samples. 50 µL of samples and standards were sequentially added to 96‐well plates, incubated at 37 °C for 30 min, and use a microplate reader (Molecular Devices) to measure the absorbance at 450 nm.

### Statistical Analysis

Statistical analysis was performed using GraphPad Prism 8.0 (GraphPad Software). The data are presented as the mean ± SD, showing continuous normal distribution. Two sample groups were analyzed using two‐tailed Student's *t*‐test. Multiple groups were analyzed using one‐way or one‐way ANOVA. Differences were considered statistically significant at *p* < 0.05. *p < 0.05, **p < 0.01, and ***p < 0.001.

## Conflict of Interest

The authors declare no conflict of interest.

## Author Contributions

W.C., Y.J., and Y.Z. conceived this research. W.C. and J.Z. conducted most experiments with the help of Y.N., Y.W., and L.X. W.C., Y.Y., and L.H. performed bioinformatic analysis. W.C. and J.Z. wrote this paper. W.C. and Y.C. analyzed the data. W.C. and Y.J. revised the document. All authors read and approved the final manuscript.

## Supporting information

Supporting Information

Supplemental Video 1

## Data Availability

The data that support the findings of this study are available from the corresponding author upon reasonable request.
