# Supplementary material for: Mitochondrial Transfer Regulates Cell Fate Through Metabolic Remodeling in Osteoporosis
Source: Adv Sci (Weinh). 2022 Dec 11;10(4):2204871. doi: 10.1002/advs.202204871 (PMC9896036; doi:10.1002/advs.202204871)
Supplement: Supplementary file 1 — Supporting Information [file ADVS-10-2204871-s002.pdf]

Supplementary Video

Supplementary Video 1. Mitochondria transfer from macrophages to MSCs. Related to Figure 1. Mitotracker Deep Red (red signal) labeled macrophages were co-cultured with Mitotracker Green (green signal) labeled MSCs. Nuclei were labeled with Hoechst 33342 (blue signal). Scale bar, 15 μm.

Supplementary Figures

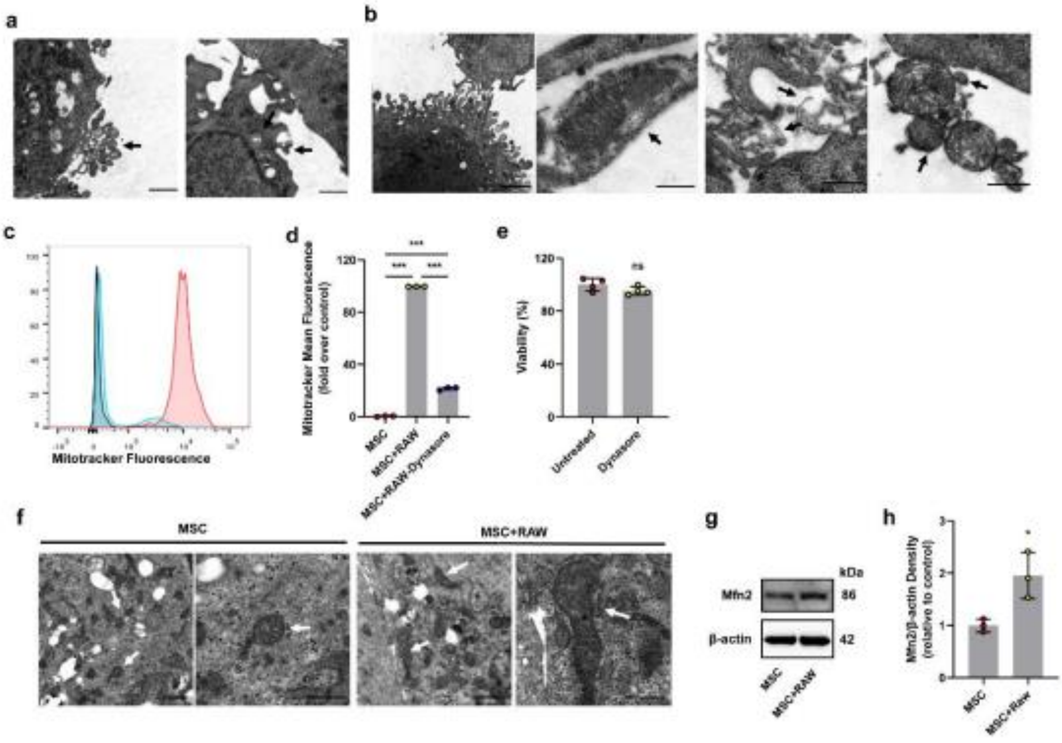

Figure S1. Mitochondria transfer from macrophages to MSCs. Related to Figure 1.

(a) Mitochondria released by macrophages. Scale bar, 2 μm; (b) Transmission electron micrographs taken after macrophage contact with MSCs (right panel). Scale bar, 2 um. Macrophages transfer mitochondria as TNTs (right second panel) and MVs (right second, third panel). Scale bar, 0.5 um (*n* = 4). (c, d) Flow cytometry analysis of the ability of MSCs to endocytose mitochondria in the absence or presence of dynasore (Dynasore+RAW) (*n* = 3);

one-way ANOVA with Tukey's post-test. (e) Relative viability of MSCs after 24 h treatment with 50 uM dynasore, as assessed by CCK8 assay for untreated (*n* = 4); two-tailed Student's t-test. (f) Transmission electron micrograph of untreated MSCs (left panel). Scale bar, 1 um. Mitochondrial morphology of untreated MSCs is short (left second panel). Scale bar, 0.5um. Transmission electron micrograph of MSCs after 24 h exposure to macrophages (right panel). Scale bar, 1 um. Mitochondrial hyperfusion was shown in MSCs (right second panel). Scale bar, 0.5 um (*n* = 4). (g, h) Western blot of Mfn2 expression in MSCs after 24 h exposure to macrophages (*n* = 3); one-way ANOVA with Tukey's post-test. Each error bar represents the mean ± SD of three independent experiments. Differences were considered statistically significant at $p<0.05$. *$p<0.05$, **$p<0.01$, and ***$p<0.001$.

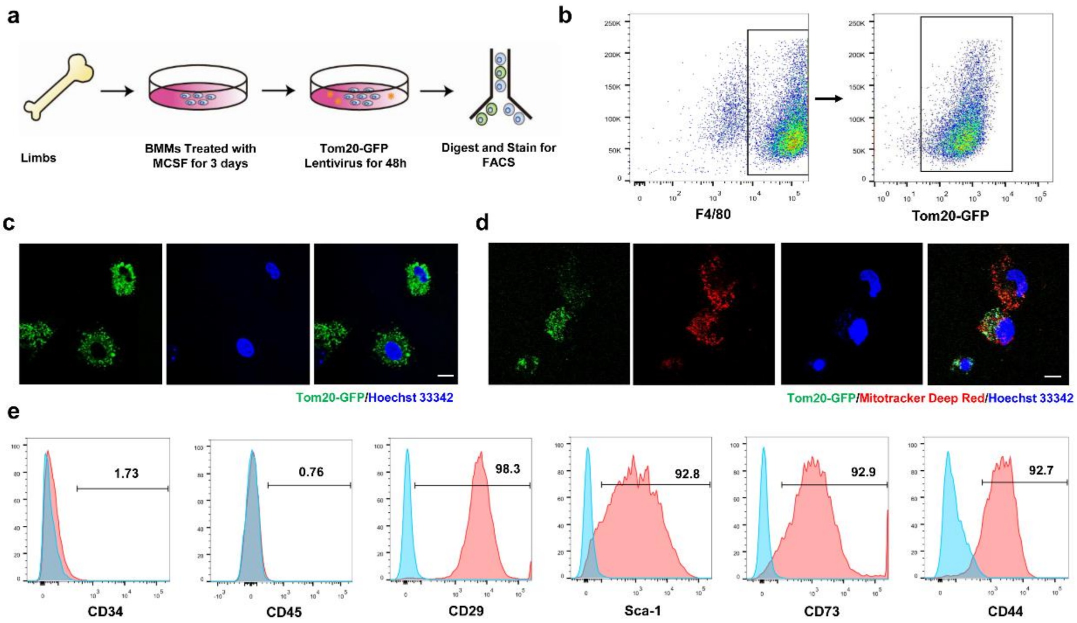

Figure S2. Analysis of macrophage mitochondrial transfer *in vivo*. Related to Figure 1.

(a-c) Schematic of obtaining Tom20-GFP expressing BMMs (a). Flow cytometry (b) and confocal microscopy analysis (c) to identify cells (*n* = 3). Scale bar, 5 µm. (d) Representative confocal microscopy pictures of Tom20-GFP (+) BMM (green) incubated with MSC for 24 hour, MSCs were previously labeled with Mitotracker Deep Red (red), and Nuclei was labeled with Hoechst 33342 (blue) (*n*=3). Scale bar, 10 µm. (*e*) Positive expression of MSC markers, including CD29, Sca-1, CD73 and CD44. There was no hematopoietic contamination of the cells as indicated by the lack of expression of CD45 and CD34 (*n* = 3). Each error bar represents the mean ± SD of three independent experiments. Differences were considered statistically significant at $p<0.05$. *$p<0.05$, **$p<0.01$, and ***$p<0.001$.

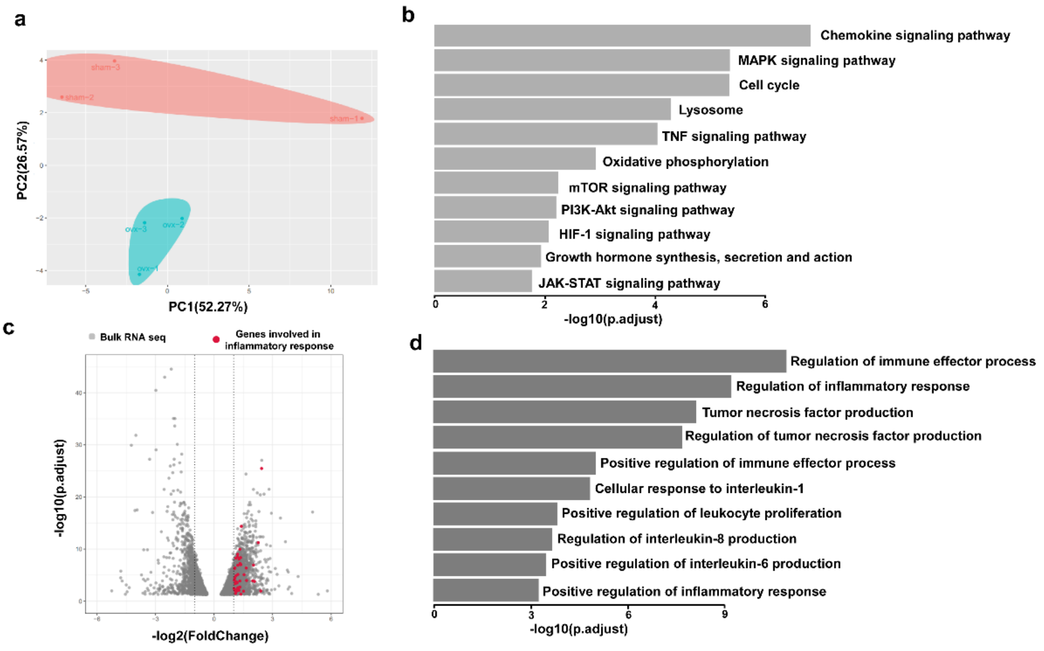

Figure S3. Bioinformatics analysis of macrophages in a mouse model of osteoporosis. Related to Figure 2.

(a) PCA based on all identified genes (*n* = 3). (b) KEGG analysis of RNA-seq data from sham or OVX-treated BMMs (*n* = 3). (c) Volcanic map of genes involved in inflammation (*n* = 3). (d) GO analysis of genes involved in inflammation (*n* = 3). Each error bar represents the mean ± SD of three independent experiments. Differences were considered statistically significant at p<0.05. *p<0.05, **p<0.01, and ***p<0.001.

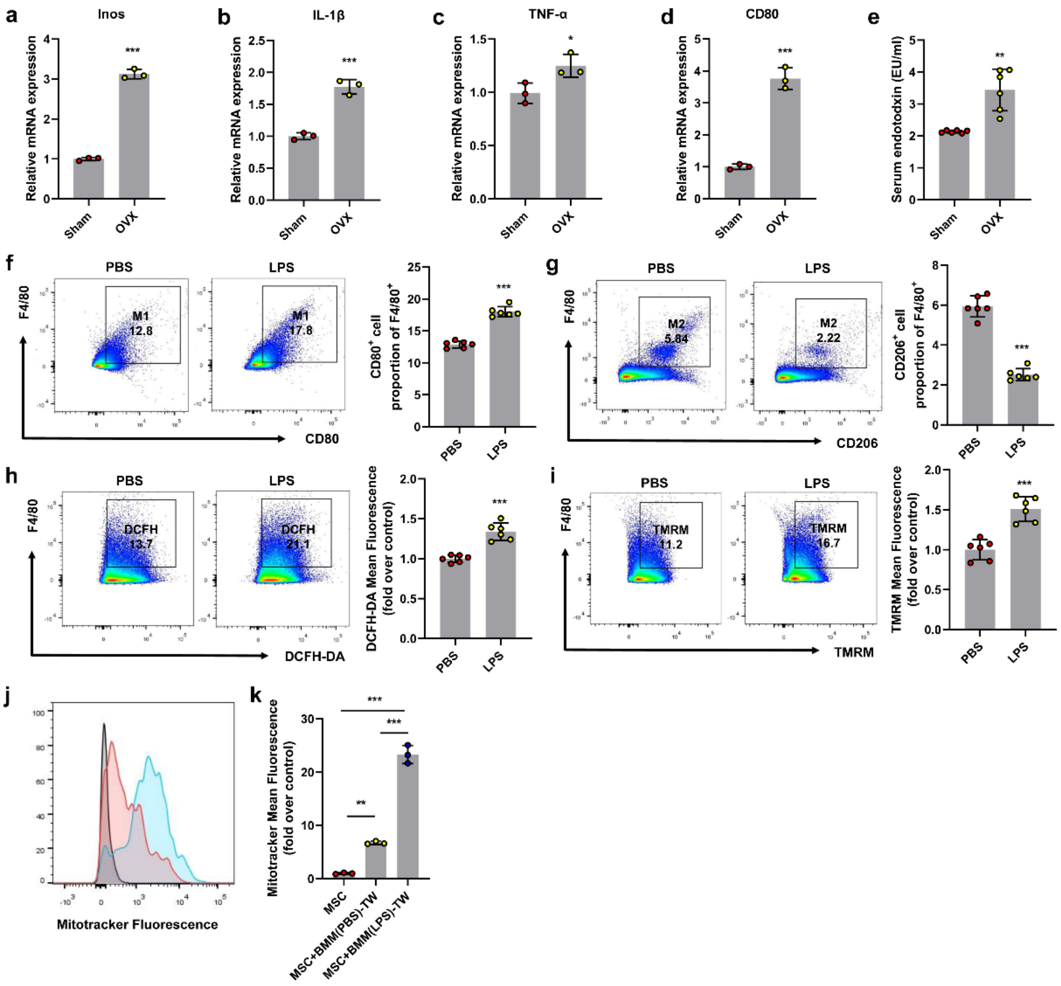

Figure S4. Endotoxin induces an increased proportion of inflammatory M1-like macrophages. Related to Figure 3.

(a-d) qPCR analysis of *Inos*, *IL-1β*, *TNF-α* and *CD80* expression in sham or OVX-treated BMMs (*n* = 3); two-tailed Student's t-test. (e) Detection of endotoxin levels in serum of sham and OVX mice (*n* = 6); two-tailed Student's t-test. (f) Flow cytometry analysis of PBS and LPS-treated mice (left) and quantification of $CD80^{+}F4/80^{+}$ macrophages in total bone marrow cells (right) (*n* = 6). (g) Flow cytometric analysis of PBS and LPS-treated mice (left) and quantification of $CD206^{+}F4/80^{+}$ macrophages in total bone marrow cells (right) (*n* = 6) two-tailed Student's t-test. (h) Macrophages from PBS and LPS-treated mice were co-treated with DCFH-DA and F4/80 for 30 min, by flow cytometry to quantify ROS, and mean fluorescence intensity was quantified as a measure of cellular reactive oxygen species production (*n* = 6). (i) Macrophages from PBS and LPS-treated mice were co-treated with TMRM and F4/80 for 30 min and then analyzed by flow cytometry to quantify membrane potential (*n* = 6); two-tailed Student's t-test. (j, k) Flow cytometric analysis (j) and

quantification of mean fluorescence intensity (k) of Mitotracker Deep Red-labeled mitochondrial internalization by MSCs in the presence of from PBS group (red line) or LPS group (blue line) macrophages. Grey line represents untreated MSCs ($n = 3$); one-way ANOVA with Tukey’s post-test. Each error bar represents the mean ± SD of three independent experiments. Differences were considered statistically significant at $p<0.05$. *$p<0.05$, **$p<0.01$, and ***$p<0.001$.

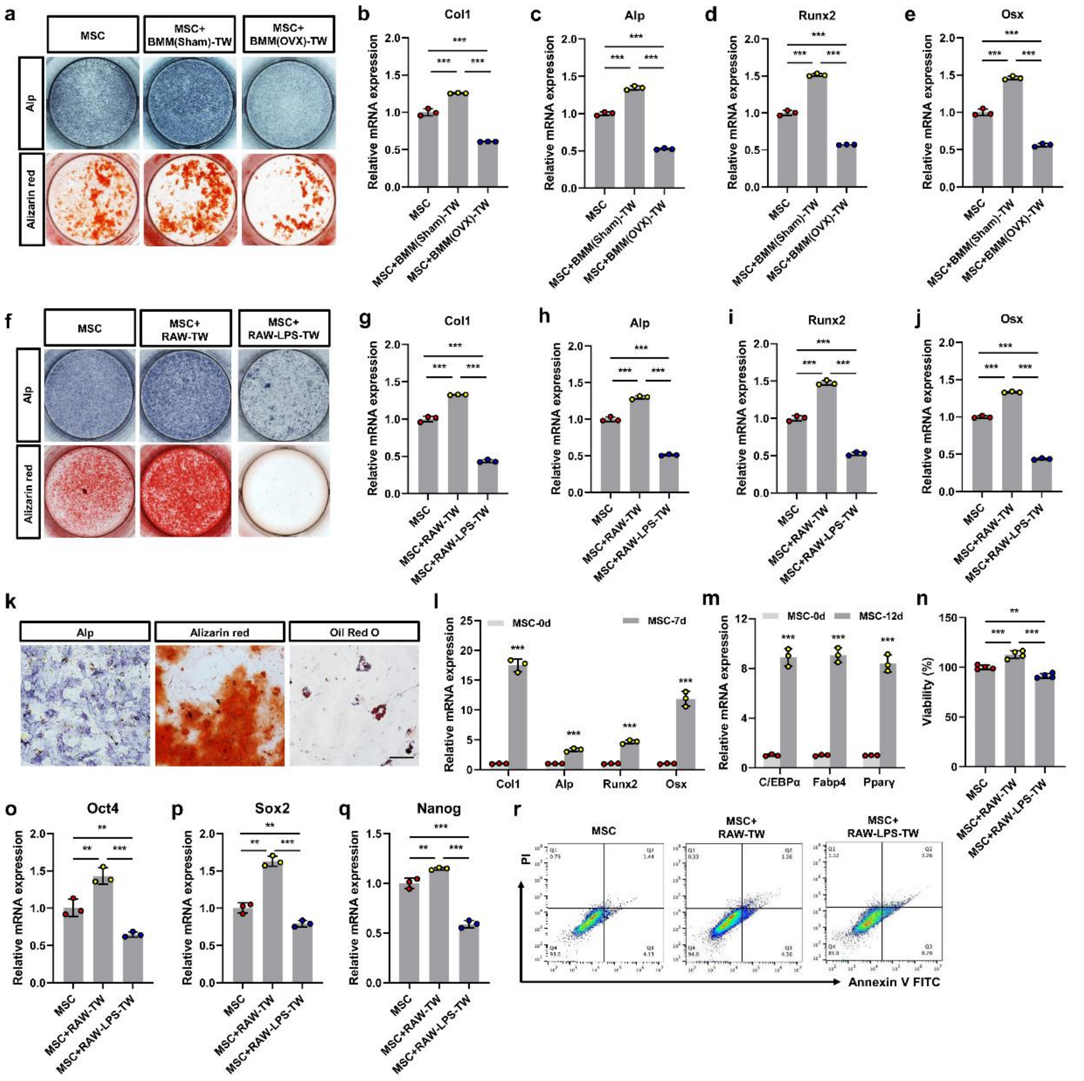

Figure S5. Mitochondria released by M1-like macrophages inhibit the osteogenic differentiation of MSCs. Related to Figure 3.

(a-e) Representative images of ALP and ARS staining in MSCs cultured alone or in the presence of sham- or OVX-derived macrophages (a) (n = 3). qPCR analysis of *Col1*, *Alp*, *Runx2* and *Osx* expression under osteogenic conditions (b-e) (n = 3); one-way ANOVA with Tukey's post-test. (f-j) Representative images of ALP and ARS staining in MSCs cultured alone or in the presence of untreated or LPS-treated macrophages (f) (n = 3). qPCR analysis of *Col1*, *Alp*, *Runx2* and *Osx* expression under osteogenic conditions (g-j) (n = 3); one-way ANOVA with Tukey's post-test. (k) Representative images of ALP, ARS and Oil Red O staining of MSCs (n = 3). Scale bar, 400 nm. (l) qPCR analysis of *Col1*, *Alp*, *Runx2* and *Osx* (n = 3); two-way ANOVA with Tukey's post-test. (m) qPCR analysis of *C/EBPα*, *Fabp4* and *Ppar-γ* (n = 3); two-way ANOVA with Tukey's post-test. (n) Cell proliferation in MSCs

cultured alone or in the presence of untreated or LPS-treated macrophages (n = 3); one-way ANOVA with Tukey's post-test. (o-q) qPCR analysis of *Oct4* (o), *Sox2* (p) and *Nanog* (q) (n = 3); one-way ANOVA with Tukey's post-test. (r) Flow cytometry analysis of cell apoptosis in MSCs cultured alone or in the presence of untreated or LPS-treated macrophages (n = 3); one-way ANOVA with Tukey's post-test. Each error bar represents the mean ± SD of three independent experiments. Differences were considered statistically significant at p<0.05. *p<0.05, **p<0.01, and ***p<0.001.

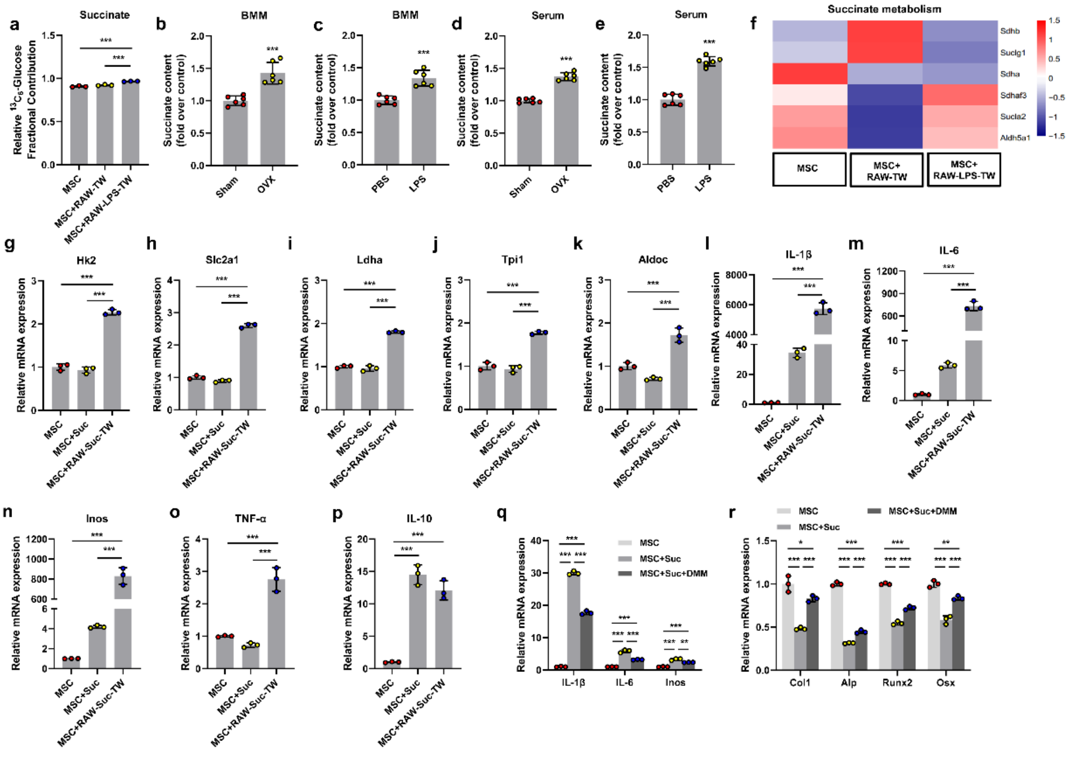

Figure S6. Intracellular accumulation of succinate in MSCs resulted in increased expression of pro-inflammatory genes. Related to Figure 5.

(a) Contribution of $^{13}C_6$-glucose to succinate (*n*=3); one-way ANOVA with Tukey's post-test. (b-e) Relative levels of succinate in BMMs (b, c) and serum (d, e) of Sham, OVX, PBS and LPS-treated mice (*n*=6); two-tailed Student's t-test. (f) Succinate metabolism genes (*n*=3). (g-p) qPCR analysis of glycolytic genes (g-k) and proinflammatory genes (l-p) in MSCs incubated with succinate or succinate-treated macrophages compared to untreated MSCs (*n*=3); one-way ANOVA with Tukey's post-test. (q) qPCR was used to analyze the proinflammatory gene expression in MSCs treated with dimethyl malonate (DMM) and succinate (*n*=3); one-way ANOVA with Tukey's post-test. (r) qPCR was used to analyze the osteogenic gene expression in MSCs treated with DMM and succinate (*n*=3); one-way ANOVA with Tukey's post-test. Each error bar represents the mean ± SD of three independent experiments. Differences were considered statistically significant at *p*<0.05. **p*<0.05, ***p*<0.01, and ****p*<0.001.

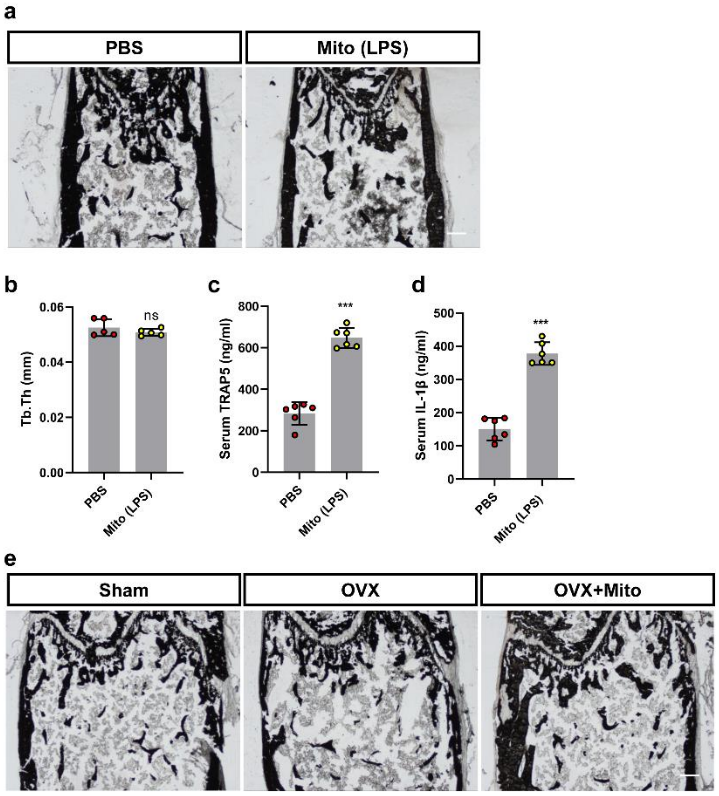

Figure S7. Skeletal phenotype of mitochondrial transfer.

(a) Representative images of Von Kossa staining of distal femoral metaphysis from PBS and Mito (LPS) mice. Scale bar, 1000 μm (*n* = 3). (b) Tb.Th analysis of mice receiving tail vein injection of LPS-treated Mito (100 μg in 100 μl PBS) or PBS (*n* = 5); two-tailed Student's t-test. (c, d) Serum TRAP5 (c) and IL-1β (d) levels in PBS and mito (LPS) mice (*n* = 6); two-tailed Student's t-test. (e) Representative images of Von Kossa staining of distal femoral metaphysis from Sham, OVX and OVX+Mito mice. Scale bar, 1000 μm (*n* = 3). Each error bar represents the mean ± SD of three independent experiments. Differences were considered statistically significant at $p<0.05$. *$p<0.05$, **$p<0.01$, and ***$p<0.001$.

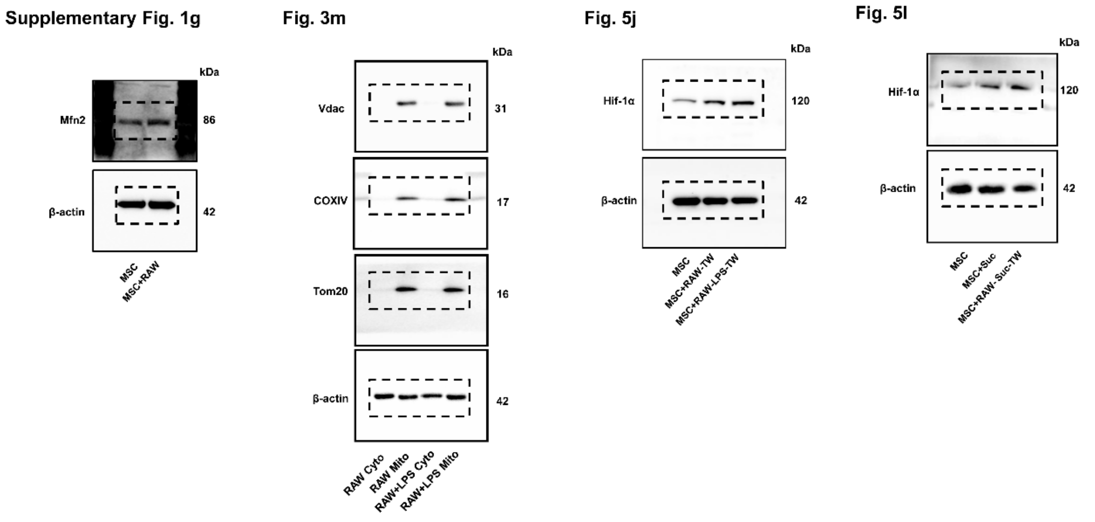

Figure S8. Original Western blot images used in main figures.

Supplementary Table

Table S1. Primer Sequences

Gene	Forward Sequence（5’-3’）	Reverse Sequence（5’-3’）
Inos	GTTCTCAGCCCAACAATACAAGA	GTGGACGGGTCGATGTCAC
IL-1β	GCAACTGTTCCTGAACTCAACT	ATCTTTTGGGGTCCGTCAACT
TNF-a	TGTCTCAGCCTCTTCTCATT	TGATCTGAGTGTGAGGGTCT
CD80	ACCCCCAACATAACTGAGTCT	TTCCAACCAAGAGAAGCGAGG
IL-10	GCTCTTACTGACTGGCATGAG	CGCAGCTCTAGGAGCATGTC
IL-6	ATAGTCCTTCCTACCCCAATTTCC	GATGAATTGGATGGTCTTGGTCC
Hif1-α	ACCTTCATCGGAAACTCCAAAG	ACTGTTAGGCTCAGGTGAACT
Hk2	TGATCGCCTGCTTATTCACGG	AACCGCCTAGAAATCTCCAGA
Slc2a1	CAGTTCGGCTATAACACTGGTG	GCCCCCGACAGAGAAGATG
Ldha	TGTCTCCAGCAAAGACTACTGT	GACTGTACTTGACAATGTTGGGA
Tpi1	CCAGGAAGTTCTTCGTTGGGG	CAAAGTCGATGTAAGCGGTGG
Aldoc	AGAAGGAGTTGTCGGATATTGCT	TTCTCCACCCCAATTTGGCTC
Col1	TGGCAAAGACGGACTCAAC	GGCAGGAAGCTGAAGTCATAA
Alp	CCAACTCTTTTGTGCCAGAGA	GGCTACATTGGTGTTGAGCTTTT
Runx2	ATTACAGATCCCAGGCAGGCA	CAGAAGTCAGAGGTGGCAGTGT
Osx	CAACCTGCTAGAGATCTGAG	TGCAATAGGAGAGAGCGA
Oct4	GGCTTCAGACTTCGCCTCC	AACCTGAGGTCCACAGTATGC
Sox2	GCGGAGTGGAAACTTTTGTCC	CGGGAAGCGTGTACTTATCCTT
Nanog	TCTTCCTGGTCCCCACAGTTT	GCAAGAATAGTTCTCGGGATGAA
C/EBPα,	CAAGAACAGCAACGAGTACCG	GTCACTGGTCAACTCCAGCAC
Fabp4	AAGGTGAAGAGCATCATAACCCT	TCACGCCTTTCATAACACATTCC
Ppar-γ	TCGCTGATGCACTGCCTATG	GAGAGGTCCACAGAGCTGATT
β-actin	CATCCGTAAAGACCTCTATGCCAAC	ATGGAGCCACCGATCCACA
